# An Ecological Panel Analysis of Trends in the Geographic Disparities of the Certified Nurse and Certified Nurse Specialist in Japan from 1996 to 2022

**DOI:** 10.3390/nursrep16010025

**Published:** 2026-01-15

**Authors:** Noriko Morioka, Tomoko Tamaki, Kunihiko Takahashi

**Affiliations:** 1 Department of Epidemiology and Biostatistics, National Institute of Public Health, Wakō 351-0197, Saitama, Japan; 2 Juntendo Hospital, Bunkyo-ku 113-0033, Tokyo, Japan; t.tamaki.oo@juntendo.ac.jp; 3 Department of Biostatistics, M&D Data Science Center, Institute of Science Tokyo, Bunkyo-ku 113-8510, Tokyo, Japan; kunihikot.dsc@tmd.ac.jp

**Keywords:** nurse workforce, advanced practice nurse, advanced practice nursing, geographic distribution, panel data analysis, ecological study

## Abstract

**Background/Objectives**: Japan introduced a certification system for Advanced Practice Nursing Workforce (APNW) in 1996. The Japanese Nursing Association formally certified two types of the APNW: Certified Nurses (CNs) and Certified Nurse Specialists (CNSs). Little is known about the geographic distribution of CNs and CNSs. **Methods**: We conducted an ecological panel analysis using prefecture-level data from 1996 to 2022. To assess the degree of inequality of CN and CNS among prefectures, we calculated the Gini overall coefficients, as well as those by categories of CN and CNS, number of hospitals, number of hospital doctors, and hospital nurses. Using data available from 2000 to 2017, we examined factors associated with CN and CNS density through fixed-effects panel data analyses of log-transformed overall and category-specific densities. **Results**: During the study period, the number of CNs and CNSs consistently increased, and geographic disparities in their distribution decreased until around 2010. After 2010, however, geographic disparities in prefectures with persistently low CN and CNS densities persisted without significant change. For overall CN and CNS density, significant associations were observed with population aging, per capita income, hospital density, hospital doctor density, hospital nurse density, and study year, whereas hospital nurse wages showed a positive but not statistically significant association. When stratified by clinical category, the directions of associations for several regional factors varied; however, hospital nurse density and hospital nurse wages tended to be positively associated with CN and CNS density in most categories. **Conclusions**: This study highlighted the need for targeted strategies to increase CN and CNS numbers specifically in prefectures with persistently low densities, tailored to each clinical category.

## 1. Introduction

Globally, geographic inequality in the distribution of the nursing workforce across nations and subnational levels has been identified [1]. As global population needs and complex and diverse health care demands grow, the Advanced Practice Nursing workforce (APNW) [2], namely Clinical Nurse Specialists or Nurse Practitioners (NPs), are expected to contribute to improving the quality of the nursing team and patient outcomes in various settings, clinical areas, and the target population [3,4,5,6] mainly through five main practices: direct comprehensive care, support of systems, education, research, and publication and professional leadership [7,8,9,10,11]. Although APNWs were expected to be equally distributed in the nursing workforce, research from the US [12,13,14,15,16,17,18,19,20], the UK [21,22], and Canada [23] documented the unequal distribution of APNWs. APNWs in primary care [12,13,14], mental health care [15,16], oncology [17,18], otolaryngology [19], and dermatologic care [20] were higher in urban than in rural areas but higher in areas with lower income and lower physician populations, which suggests that APNWs substituted for the shortage of physicians. The APNW distribution also affects the policy in that the growth rate is slower in areas with restrictive NP practice regulations than in areas with full practice authority for NPs [21,22]. However, more knowledge of the factors associated with APNW distribution is necessary to achieve equal accessibility.

Japan introduced a certification system for APNWs in 1996. The Japanese Nurse Association (JNA) formally certifies two types of nurses as part of the APNW: Certified Nurses (CNs) require over 600 h in addition to more than five years of clinical experience, and Certified Nurse Specialists (CNSs) require a master’s degree in addition to more than five years of clinical experience (JNA) [23]. Although the global definition of advanced practice nurse (APN) requires at least a master’s degree, both CN and CNS have functioned as providers of advanced practice nursing in Japan. This reflects the historical context in which the APN certification system was established, when nurses with a bachelor’s degree were scarce. CNs have been divided into two courses since 2020: an existing course (course A) and a new course (course B) that allows the delivery of specific medical care, such as tracheostomy tube replacement or bladder fistula catheter replacement. There are 3155 CNSs in 14 fields and 28,261 CNs (20,710 CNs with course A in 21 fields, 2550 CNs with course B in 19 fields, and 5001 CNs in administration) as of December 2022 (see Supplementary File) [24,25,26]. They have contributed to implementing evidence-based nursing practice [27] and patient outcomes [28,29,30] as APNWs, and both are positioned equivalently within the national fee schedule, with identical financial incentives. The unequal regional distribution of hospital nurses has been an issue in Japan [31,32], as it has in other countries. Prior studies have predominantly focused on the geographical distribution of general registered nurses, often without distinguishing between general practitioners and specialized professionals like CNs and CNSs. Consequently, little is known about the geographical distribution of APNW, such as CNs and CNSs, and the specific regional factors associated with their supply.

Currently, the APNWs in Japan have been reformed and continue to be under discussion. In 2015, Article 37-2 of the Act on Public Health Nurses, Midwives, and Nurses introduced a new nursing system that allows additional training to deliver specific medical treatments. Following this legislative amendment, the JNA reorganized the CN certification framework in 2020. In addition to the CN reform, the JNA held discussions with stakeholders and lobbied the ruling party to establish an NP system. In this discussion on reforming the CN and CNS certification system, the perspective of achieving equal accessibility for CNs and CNSs based on evidence should be included.

Therefore, we aimed to examine the trend in the degree of geographic distribution of CNs and CNSs overall and by clinical category over 27 years from 1996, when the certification system was introduced, to 2022 in 47 prefectures (subnational level) in Japan, which are designated as tertiary healthcare planning areas responsible for coordinating and completing advanced and emergency medical care, including tertiary emergency services. We also investigated the regional factors associated with CN and CNS density per population.

## 2. Materials and Methods

### 2.1. Background of Certification System of Advanced Practice Nursing

A nurse in Japan must acquire a nursing license (national qualification) from Japan’s Ministry of Health, Labour, and Welfare (MHLW) to provide medical treatment or assist in medical care for injured and ill persons or puerperal women (Article 5 of the Act on Public Health Nurses, Midwives, and Nurses). Although the number of nurses has increased annually and 1.73 million nurses were working as of 2020, the shortage of the nursing workforce has been identified and it is spreading in this aging society [31,33]. In 1987, the discussion on the certification system for APNW by the MHLW began, and the JNA introduced the certification system of the CNS in 1996 and the CN in 1997. Initially, an APN needed a minimum of a master’s degree; however, as of 1996, there were only eight universities or colleges of nursing with master’s programs in Japan, and nurses with a bachelor’s degree were scarce. Therefore, the MHLW and JNA introduced a CN system which did not require a bachelor’s degree in nursing and only 600 h of education. Both CNs and CNSs provide advanced practice nursing. CNSs are expected to play a role in practice, consultation with care providers, coordination between health and social care professionals, ethical coordination, education and research, and excellence in nursing practice. CNs are expected to play a role in the high standards of nursing practice, instruction, and consultation with nursing professionals and others, with a high level of clinical reasoning and judgment of pathological conditions. The CN with course A will be closed in FY 2026, and CN training will be transformed to course B only in FY 2026. The total cost of CN or CNS is estimated to be more than 2 million yen, as tuition fees and certification examination fees will cost approximately 1~1.5 million yen, and moving and accommodation costs for commuting to the training institute will be additional. CNS and CN candidates can take out scholarships from the JNA, prefectural grants, scholarships provided by the respective graduate schools, and financial support from the hospital where they work; however, few can cover the full amount, and any shortfall must be met at their personal expense.

Apart from the CN and CNS certification system by the JNA, the NP certification system exists in two different organizations: the Japanese Organisation of Nurse Practitioner Faculties (JONPF) introduced NP in 2011 and the Japan Association of Nursing Programs in Universities (JANPU), which was developed as an original educational curriculum for NP in primary care nursing in 2015 [34]. Since 2020, JNA, JONPF, and JANPU have discussed the NP certification system. Because national or local laws do not recognize these yet, we did not include NPs in the analysis

### 2.2. Design

We conducted an ecological panel analysis using prefecture-level (subnational) data in Japan from 1996 to 2022.

### 2.3. Variables and Data Sources

#### 2.3.1. Outcome

The numbers of CNs and CNSs were used as the outcome variables in 47 prefectures. A total of 31 fields of CN (21 fields in CNs with course A, 19 fields in CNs with course B, and CN in administration) and 14 fields of CNS were categorized into nine categories: (1) primary care, (2) infection control, (3) critical care, (4) wound, ostomy, and continence (WOC), (5) gerontological nursing, (6) chronic care, (7) cancer care, (8) child, family, and reproductive health, and (9) administration (see Supplementary File). The categorization was performed by two nursing researchers with experience in clinical practice. Data on prefecture-level numbers in each field of CNS or CN data were obtained from the JNA website [23,24,26], and we combined the data into the dataset.

#### 2.3.2. Regional Characteristics

As socioeconomic variables, we used the percentage of the population aged 65 and older [35], and average income per capita [36]. Average income per capita is an indicator of income disparities among prefectures, which is calculated as total income, including prefectural employee wages and salaries, financial assets, and corporate income, by total population [36].

For medical and nurse-related variables, we used the number of doctors and nurses working in hospitals obtained from Survey Medical Institute [37], and the average hourly salary of nurses obtained from the Basic Survey on Wage Structure [38].

### 2.4. Statistical Analysis

First, we used a bar chart to show the trend in the national total number and nine categories of CNs and CNSs from 1996 to 2022. We calculated the median and interquartile range (IQR) of the prefecture characteristics. To assess the degree of inequality of CNs and CNSs among prefectures, we calculated the Gini coefficients of the total and by categories of CNs and CNSs, number of hospitals, number of hospital doctors, and hospital nurses.

The Gini coefficient was calculated using the following equation: [39]

(1) $$G=1-\sum_{i=0}^{n}\left(Y_{i+1}+Y_{i}\right)\times \left(X_{i+1}+X_{i}\right)$$

where

*n* = total number of prefectures;

*Y_i_* = cumulative percentages of CNs and CNSs or other medical resources in the prefecture;

*X_i_* = cumulative percentage of the population belonging to the prefecture.

Next, we calculated the density of CNs and CNSs in total and in nine categories: number of hospitals, number of hospital doctors, and number of hospital nurses per 10,000 people. To show the trend in the distribution of CN and CNS density, we created box plots from 1996 to 2022 and line plots of the highest and lowest CN and CNS density prefectures as of 2022.

To investigate the factors related to CN and CNS density, we conducted a panel data analysis with fixed effects, predicted the log of overall CN and CNS density, and stratified it into nine categories using data from 2000 to 2017, owing to limited data availability. Prefecture–year observations with zero CN and CNS density were retained in the analyses to reflect the true absence of the workforce. For log transformation, a small constant (0.1) was added to CN and CNS density values for all observations (i.e., log[x + 0.1]) to allow inclusion of zero observations. As a sensitivity analysis, we repeated the analysis using an alternative constant of 1 (i.e., log[x + 1]). The independent variables in the model were selected based on a literature review [31,32]. The CN and CNS density, hospital densities, number of hospital doctors, number of hospital nurses, average income per capita, and hourly salary of hospital nurses were natural log-transformed because of their skewed distributions.

(2) $$\begin{array}{l} \log\left({\mathrm{number of CN and CNS density}}_{it}\right)=\beta_{0}+\beta_{1}{\left(\mathrm{percentage of}\;65+\mathrm{population}\right)}_{it}\\ \;+\beta_{2}\log{\left(\mathrm{average income per capita}\right)}_{it}\\ \;+\beta_{3}\log{\left(\mathrm{hospital density}\right)}_{it}+\beta_{4}{\log(\mathrm{hospital doctor density})}_{it}\\ \;+\beta_{5}{\log(\mathrm{hospital nurse density})}_{it}+\beta_{6}{\log(\mathrm{average hourly salary of nurse})}_{it}\\ \;+\beta_{7}{\left(\mathrm{year}\right)}_{it}+u_{i} \end{array}$$

where

*i* = prefecture;

*t* = year;

*u_i_* = prefecture-specific intercept.

When the independent variables were logarithmic transformation or the percentage of the population aged 65 and older, a 1% change in the independent variables was associated with a β_k_% change in the CN and CNS density. When the independent variable was year, which was not a logarithmic transformation, one year change was associated with a 100 × [exp(β_7_ − 1)]% change in CN and CNS density [40]. Significance level was set at *p* < 0.05. All experiments were performed using Stata ver. 18.

### 2.5. Ethical Consideration

Individual data were not included. This study was conducted in accordance with the Declaration of Helsinki (https://www.wma.net/policies-post/wma-declaration-of-helsinki/, accessed on 3 December 2023) and the study protocol was approved by the National Institute of Public Health Ethics Review Board (No. NIPH-TRN#25001, 9 June 2025).

## 3. Results

**Figure 1:**
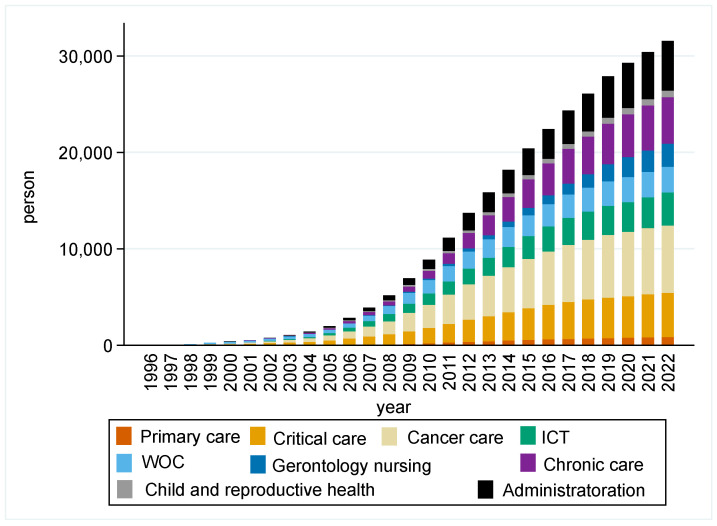
Trend in the total number of CNs and CNSs from 1996 to 2022 in Japan.

The nationwide total number of CNs and CNSs increased from 6 in 1996 to 31,538 in 2022 (Figure 1), and the median (IQR) of CNs and CNSs in 47 prefectures increased from 0 (0–0) to 429 (301–682) (Table 1). Every five-year trend in the regional characteristics is shown in Table 1. The median population declined from 1790 thousand persons in 1996 to 1610 thousand in 2022; as such, the median of percentage of the population aged 65 and older increased from 17.6% to 31.0%. The number of hospitals remained almost unchanged, whereas the number of hospital doctors and nurses increased.

**Figure 2:**
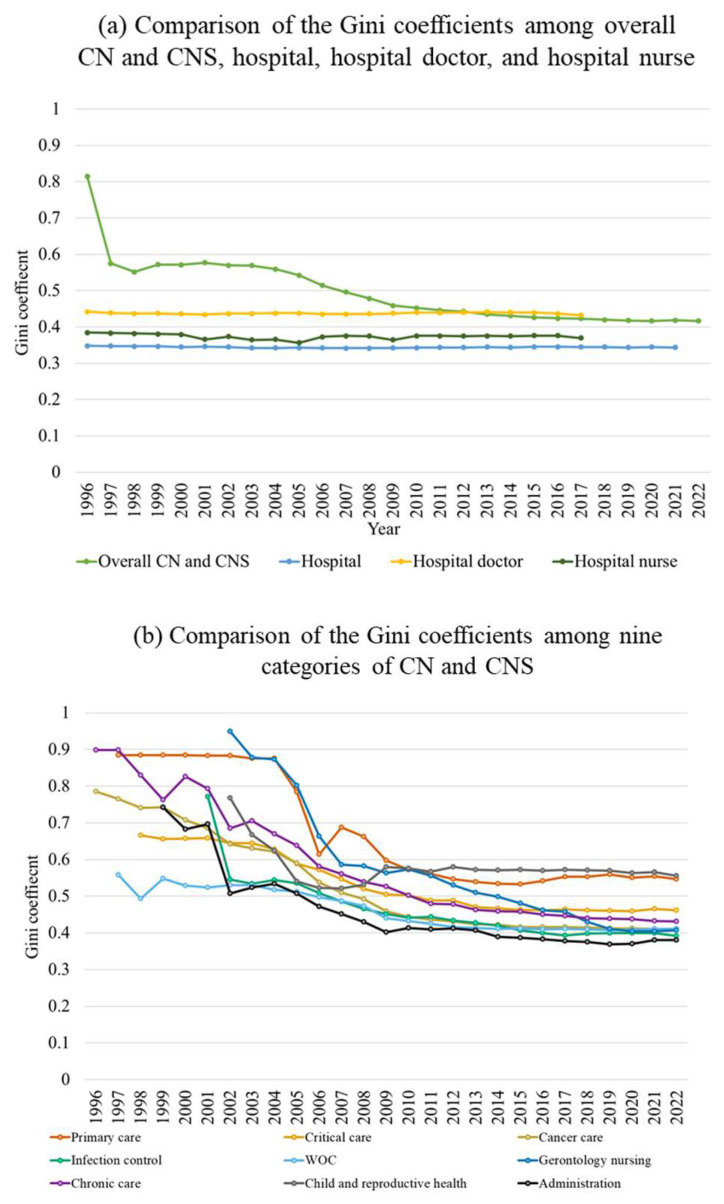
Comparison between the Gini coefficients for CN and CNS and medical resources. (**a**) Comparison of the Gini coefficients between overall CN and CNS, hospital, hospital doctor, and hospital nurse; (**b**) comparison of the Gini coefficients between nine categories of CN and CNS.

The Gini coefficient of overall CNs and CNSs declined markedly from 0.8 in 1996 to 0.4 by 2010, after which it plateaued and remained at approximately 0.4 through 2022 (Figure 2a). In descending order, the Gini coefficients in 2017 were as follows: hospital doctors, overall CNs and CNSs, hospital nurses, and hospitals (Figure 2a). Trends in Gini coefficients by CNs and CNSs clinical category showed that the Gini coefficients decreased in all categories. In 2022, the category with the largest Gini coefficient was pediatric and primary care at 0.6, followed by oncology and chronic care. The largest margin of difference in the Gini coefficients was in elder care, which decreased from 0.9 in 2002 to 0.4 in 2022 (Figure 2b).

**Figure 3:**
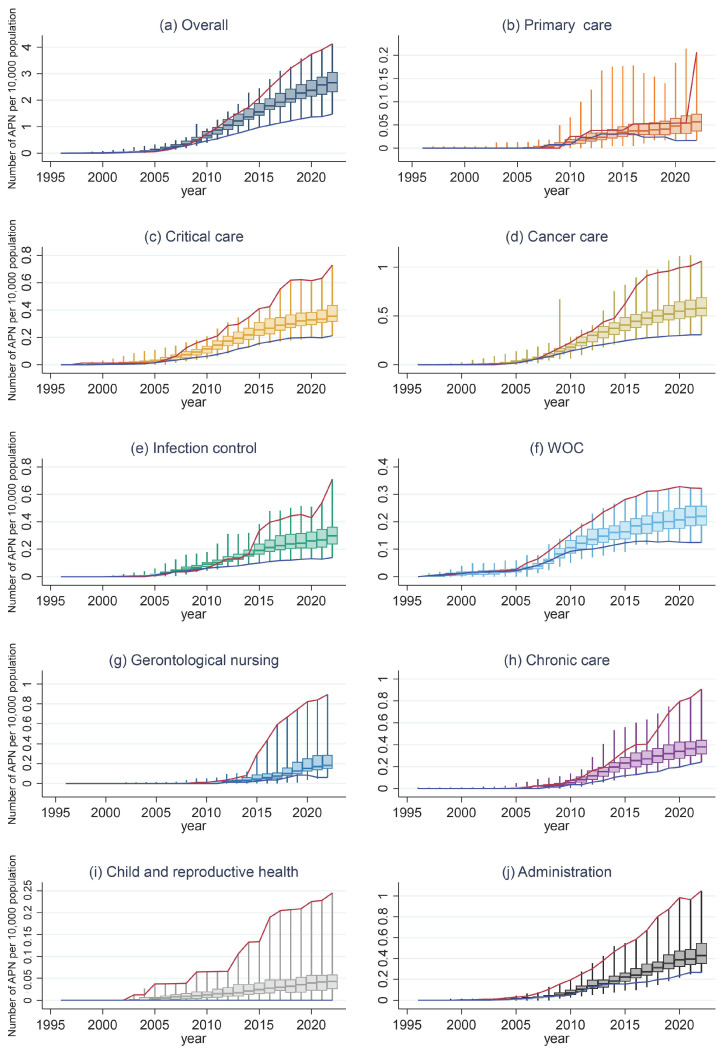
Trends in the distribution of the CNs and CNSs per 10,000 capita. (**a**) Overall; (**b**) primary care; (**c**) critical care; (**d**) cancer care; (**e**) infection control; (**f**) wound, ostomy, and continence (WOC); (**g**) gerontological nursing; (**h**) chronic care; (**i**) child and reproductive health; (**j**) administration. The red line shows the annual trends for the prefectures with the highest density of CNs and CNSs as of 2022, and the blue line shows the same for the prefectures with the lowest density as of 2022.

With the trend in the distribution of CN and CNS density, the means of overall density and each clinical category of CN and CNS density have increased annually, and the ranges of overall density and each clinical category of CN and CNS density have spread widely (Figure 3). For overall and critical care, WOC, gerontological nursing, child and reproductive health, and administration, the annual trends for the prefectures with the highest density of CNs and CNSs as of 2022 always placed them at the top of the list (Figure 3a,c,f,g,i,j). For the primary care, cancer care, infection control, and chronic care categories, the prefectures with the highest CN and CNS density as of 2022 were at the median level in 2021, 2013, and 2017, respectively, and then switched to the upper tier (Figure 3b,d,e,h).

**Figure 4:**
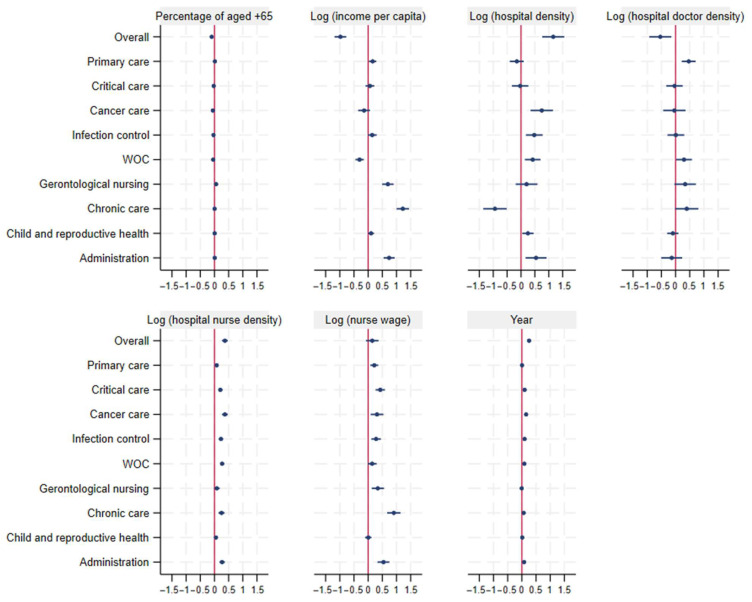
Coefficients and 95% confidence intervals of panel data fixed-effect models for the log of the overall CN and CNS density (per 10,000 person) and by nine categories.

Regarding regional factors associated with overall CN and CNS density, statistically significant associations were observed for the percentage of the population aged 65 years and older (coefficient −0.10, 95% CI −0.12–−0.08), per capita income (−0.98, 95% CI −1.19– −0.7), hospital density (1.16, 95% CI 0.69–1.56), hospital doctor density (−0.54, 95% CI −0.93–−0.15), hospital nurse density (0.37, 95% CI 0.27–0.47), and study year (0.26, 95% CI 0.24–0.27), whereas hospital nurse wages showed a positive but non-significant association (0.14, 95% CI −0.08–0.37) (Figure 4). When stratified by clinical category, the directions of associations for the percentage of the population aged 65 years and older, income per capita, hospital density, and hospital doctor density varied across categories. Despite this heterogeneity, hospital nurse density and the hourly wage of hospital nurses were positively associated with CN and CNS density in most clinical categories. Sensitivity analyses using an alternative constant of 1 yielded substantively similar results.

## 4. Discussion

This ecological panel analysis at the prefecture level revealed that, despite a continuous increase in the absolute number of CNs and CNSs, geographic disparities in their distribution narrowed until approximately 2010 but subsequently plateaued, remaining at a level comparable to that observed for hospital doctors.

The disparity between prefectures was eliminated for about 10 years after 1996, when the CN and CNS certification system started, as the number of people increased; however, the extent of the disparity did not decrease in the following decade, despite an increase in the overall number of people. The fact that the disparity has not been eliminated despite the increase in numbers is consistent with the finding of disparity among hospital nurses in Japan [31,32]. One possible explanation is that the distribution of CNs and CNSs depends on the number of hospital beds per population, which itself exhibits substantial geographic variation [32]. Further studies are needed to gain a deeper understanding of the structural and contextual factors underlying the persistent geographic disparities in the distribution of CNs and CNSs.

The present study also revealed that lower CN and CNS density prefectures were consistent over 27 years, and this trend was observed both overall and when stratified by clinical category. This is consistent with a study on NP disparity in the US, which showed that the growth rate in areas with lower NP density was slower than that in areas with higher NP density [14]. A lack of financial support is a barrier to nurses’ access to APN training [41,42]. In Japan, some prefectures have policies for CN development, with hospitals subsidizing nurses’ salaries at 2 million yen per year to fill vacancies through training and around 1 million yen in tuition fees for candidates, and the JNA also provides for subsidies for nurses. However, these are not specific to areas with low CN and CNS densities. Therefore, there is a need to support the growth of CNs and CNSs, specifically in areas with low CN and CNS density.

We also identified regional factors associated with CN and CNS density. Prefectures with lower per capita income tended to have higher overall CN and CNS density, which is consistent with previous studies conducted in the United States and Canada [12,13,19,20]. However, the direction and magnitude of these associations varied across clinical categories, suggesting that the mechanisms linking regional socioeconomic conditions and CN/CNS distribution may differ by specialty. This heterogeneity highlights the need for more detailed, category-specific analyses in future research. Although regions with a higher proportion of older adults would generally be expected to have greater needs for advanced practice nursing services, we did not observe a clear positive association between the percentage of the population aged 65 years and older and CN and CNS density. This finding may indicate a potential mismatch between population-level needs and the current distribution of the CN and CNS workforce, rather than an absence of need in these regions.

For medical- and nurse-related variables, higher hospital nurse density and higher hospital nurse wages were consistently associated with higher CN and CNS density, both overall and across most clinical categories. Because eligibility for CN and CNS certification requires at least five years of clinical experience as a registered nurse, regions with a larger hospital nursing workforce represent a larger pool of nurses who meet the qualification requirements for CN and CNS training. This finding is therefore plausible and underscores the importance of the size of the experienced nursing workforce in shaping regional differences in CN and CNS density. The positive association between higher hospital nurse wages and CN and CNS density is also consistent with prior evidence indicating that higher wages for generalist nurses are associated with better retention of hospital nurses within a region [9]. This suggests that wage levels may influence CN and CNS density indirectly by sustaining a larger pool of experienced nurses eligible for advanced practice certification, rather than through direct financial incentives linked to CN or CNS qualifications. Indeed, in Japan, wages do not generally reflect CN or CNS credentials; a nationwide survey conducted by the Japanese Nursing Association in 2022 reported that only 12.7% of CNSs experienced an increase in salary as a result of obtaining CNS certification [43]. Future research should therefore examine how broader working conditions, such as wages for generalist nurses, defined professional roles, and additional financial incentives specific to CNs and CNSs, may influence their regional distribution and density.

This study had some limitations. First, the availability of data on the number of CN or CNS educational affiliations by prefecture is limited. Although information on the number of training sessions has been noted in previous studies as a factor related to the number of nurses, the data were not available for this study. The number of CN or CNS education affiliations by prefecture opened only in 2023, and there were no available data from 1996 to 2022. In addition, in some CN or CNS training schools, training programs are only offered every two or three years, making it difficult to construct a year-by-year panel dataset over time. A data collection system is required over time to determine the status of the number of training sessions by prefecture.

Second, due to data availability constraints, analyses of factors associated with CN and CNS density were restricted to the period from 2000 to 2017. Consequently, regional factors related to APNW distribution could not be examined for the entire study period from 1996 to 2022. In addition, to include prefecture–year observations with zero CN and CNS density in the regression models, a small constant was added prior to log transformation. While this approach allowed us to retain information on the true absence of CNs and CNSs, it requires caution in interpreting the estimated coefficients, as they reflect associations with log-transformed density values rather than direct absolute changes in CN and CNS density.

Third, there is an issue regarding the appropriate geographical unit of analysis. Although secondary healthcare areas represent the primary units for general inpatient care delivery in Japan, this study used prefectures as the unit of analysis. This choice was driven by data availability, as nationally standardized and longitudinal data on CNs and CNSs and key covariates were available only at the prefectural level throughout the study period. Moreover, prefectures are designated as tertiary healthcare planning and provision areas responsible for coordinating advanced and emergency medical care, including tertiary emergency services, and serve as key administrative units for healthcare workforce planning and policy implementation. Nevertheless, the misalignment between service delivery areas and administrative units may have masked within-prefecture variation and attenuated observed associations. Therefore, the findings should be interpreted as reflecting broader regional patterns rather than local service-level disparities. Future studies using secondary healthcare areas as the unit of analysis are warranted once appropriate data become available.

## 5. Conclusions

This study identified the 27-year trends in CN and CNS density and geographical distribution at the sub-national level in Japan as well as relevant regional characteristics. Although the number of CNs and CNSs increased across all clinical categories over the study period, geographic inequality decreased until around 2010 but plateaued thereafter, with the gap between high- and low-density prefectures persisting or even widening. In addition, higher hospital nurse density and hospital nurse wages were positively associated with CN and CNS density in overall analyses and across most clinical categories, suggesting the importance of the hospital nursing workforce and working conditions in shaping regional distribution. Moving forward, policies to further reduce regional disparities, such as implementing financial incentives and creating supportive work environments that enable CNs and CNSs to fully utilize their roles, are needed to increase CN and CNS density in areas where it is currently low, and thus achieve equitable access to advanced practice nursing care.

## Figures and Tables

**Table 1:** Trend in the number of CNs and CNSs and prefecture characteristics from 1996 to 2022 in 47 prefectures in Japan.

	Year																	
	1996			2001			2006			2011			2016			2022		
	Median	IQR		Median	IQR		Median	IQR		Median	IQR		Median	IQR		Median	IQR	
**Number of total CNs and CNSs (person)**																		
Overall	0	0	0	5	2	11	30	21	60	141	93	240	288	216	499	429	301	682
By category																		
Primary care	0	0	0	0	0	0	0	0	1	3	2	5	6	4	13	10	4	16
Critical care	0	0	0	1	0	3	7	3	16	21	16	44	43	32	79	57	40	90
Infection control	0	0	0	0	0	1	4	3	7	16	11	35	35	25	64	48	31	83
Wound, ostomy, and continence (WOC)	0	0	0	2	1	5	5	3	9	19	13	38	29	21	61	34	25	65
Cancer care	0	0	0	1	0	2	8	5	17	38	26	74	76	52	128	96	68	151
Gerontological nursing	0	0	0	0	0	0	0	0	1	3	1	5	11	6	26	37	20	65
Chronic care	0	0	0	0	0	0	2	1	5	12	8	24	42	28	77	64	38	107
Child and reproductive health	0	0	0	0	0	0	1	0	2	3	1	5	5	2	10	6	3	17
Administration	0	0	0	0	0	1	4	1	6	18	11	32	38	30	87	67	53	144
**Prefecture social and economic status**																		
Population (100 thousand persons)	17.9	11.9	28.7	17.8	11.8	28.7	17.6	11.7	28.7	17.1	11.5	28.5	16.8	11.3	28.6	16.1	10.6	27.9
Percentage of aged 65+ population (%)	17.6	15.0	18.7	19.9	17.5	21.6	22.5	19.9	23.9	24.5	22.4	26.1	28.1	26.2	29.6	31.0	29.3	33.1
Average annual income per capita (100 thousand yen) *	29.5	26.3	32.0	27.7	25.0	29.5	27.9	24.8	29.8	26.6	24.1	28.2	29.1	26.3	30.6	NA	NA	NA
Average hourly salary of hospital nurses (thousand yen)	NA	NA	NA	2.3	2.2	2.5	2.2	2.1	2.3	2.3	2.1	2.4	2.4	2.2	2.4	NA	NA	NA
**Prefecture medical resources**																		
Number of hospitals density	0.8	0.6	1.1	0.7	0.6	1.0	0.7	0.6	1.0	0.7	0.6	1.0	0.7	0.6	1.0	NA	NA	NA
Number of hospital doctors density	12.8	11.2	14.6	13.6	11.8	15.5	14.1	12.6	16.5	15.9	13.8	18.2	17.0	15.0	19.5	NA	NA	NA
Number of hospital nurses density	39.5	32.1	46.7	66.9	53.7	77.7	51.4	44.1	60.0	62.2	51.6	71.5	70.3	58.8	81.2	NA	NA	NA

* Thousand yen = USD 6.3. CN: Certified Nurses, CNS: Certified Nurse Specialists, IQR: inter quartile range, NA: not applicable. Density means figure per 10,000 populations.

## Data Availability

The datasets for the current study are available with the Japanese Nursing Association (https://www.nurse.or.jp/nursing/qualification/vision/cn/; https://www.nurse.or.jp/nursing/qualification/vision/cns/index.html; https://www.nurse.or.jp/nursing/qualification/vision/cna.html, accessed on 19 December 2023).

## Supplementary Materials

The following supporting information can be downloaded at: https://www.mdpi.com/article/10.3390/nursrep16010025/s1.

## Public Involvement Statement

There was no public involvement in any aspect of this research.

## Guidelines and Standards Statement

This manuscript was drafted against the Strengthening and Reporting of Observational Studies in Epidemiology guidelines.

## Abbreviations

The following abbreviations are used in this manuscript:

APN	Advanced Practice Nurse
APNW	Advanced Practice Nursing Workforce
CN	Certified Nurse
CNS	Certified Nurse Specialist
JANPU	Japan Association of Nursing Programs in Universities
JNA	Japan Nursing Association
JONPF	Japanese Organisation of Nurse Practitioner Faculties
MHLW	Ministry of Health, Labour, and Welfare
NP	Nurse Practitioner
