# Supplementary material for: An Ecological Panel Analysis of Trends in the Geographic Disparities of the Certified Nurse and Certified Nurse Specialist in Japan from 1996 to 2022
Source: Nurs Rep. 2026 Jan 15;16(1):25. doi: 10.3390/nursrep16010025 (PMC12844417; doi:10.3390/nursrep16010025)
Supplement: Supplementary file 1 [file nursrep-16-00025-s001.zip › Supplementary file. Details of APN in each fields.pdf]

Supplementary file. Details of Certified Nurse and Clinical Nurse Specialist in each clinical category.

Clinical Category in this Study	CN/CNS	Field	Details of CN or CNS in Each Field	Established Year
Primary and community care	CNS	Community Health Nursing	Provide a high standard of nursing care in one of the following areas: occupational health, school health, health administration, or home care, and contribute to the development of health and medical welfare in the community.	1996
		Home Care Nursing	Provide a high standard of nursing care by establishing a new care system in home care nursing and promote the coordination of existing care services. Support patients and their families who receive medical care at home to continue their daily lives where they deserve.	2012
	CN	(A) Visiting Nursing	Provide and manage self-care support and case management techniques that respect home care patients' independence. Maintain and improve quality of life at home and support self-care.	1998
		(B) Home Care	In addition to A courses' activities, B courses require knowledge and skills to change the tracheal cannula, gastroduodenal catheter, enteric and catheter, gastroduodenal button, and safely remove necrotic tissue without blood flow in treating bedsores and chronic wounds.	2019
Critical care	CNS	Critical Care Nursing	Provide a high standard of intensive care nursing for patients with high urgency or severity, support patients and their families, and coordinate among medical staff to ensure the best possible medical care.	2004
		Disaster Nursing	Considering the characteristics of disasters, provide appropriate nursing care, including mental health care, with limited human and material resources.	2016
	CN	(A) Emergency Nursing	Requires rapid lifesaving techniques and triage according to the condition of the patient, care for acute medical needs during disasters, and early intervention and support for patients and families in crisis.	1995
		(A) Intensive Care	Prevention of severe disease by predicting pathological changes in patients with life-threatening conditions, and prevention of secondary complications such as disuse syndrome and implementing early rehabilitation for recovery.	1997
		(B) Critical Care	In addition to A courses' activities, B courses require knowledge and skills to adjust the dosage of sedatives for those on ventilatory management, wean patients from ventilators, and adjust the dosage of drugs during continuous infusion.	2019
	CN	(A) Neonatal Intensive Care	Predict pathological changes in high-risk newborns to prevent serious illness, care for physiological stability and developmental promotion, and support parent–child relationships. Provide individualized care for growth and development without disability.	2001
		(B) Neonatal Intensive Care	In addition to A courses' activities, B courses require knowledge and skills to perform tracheal cannula exchange.	2019
	CN	(A) Perioperative Nursing	Safety management to minimize surgical invasion and prevent secondary complications, continuity of nursing practice in the perioperative period, and promptly respond to any patient complications and emergencies during surgery.	2003
		(B) Perioperative Nursing	In addition to A courses' activities, B courses require knowledge and skills to adjust the position of an oral or nasal tracheal tube, change the setting of the invasive positive pressure ventilator, wean the patient from the ventilator, arterial blood sampling, the dose of analgesic drugs via the epidural catheter, and the dosage of carbohydrate or electrolyte infusions during continuous infusion.	2019

	CN	(A) Pediatric Emergency Nursing	Perform rapid lifesaving techniques and triage according to the child's condition in an emergency, respond to parental anxiety or child abuse, and protect the rights of children and parents. Prevention, early detection, and triage of serious illness in critically ill settings, inappropriate childcare, or abuse.	2004
		(B) Pediatric Primary Care	In addition to A courses' activities, B courses require knowledge and skills to assess physical symptoms and change tracheal cannulas.	2019
Cancer Care	CNS	Cancer Nursing	Understand the physical and emotional distress of cancer patients and provide a high standard of nursing care from the perspective of quality-of-life to patients and their families.	1995
		Radiological Nursing	Establish a system for radiation accidents and disasters from normal times and provide a high standard of long-lasting nursing care for patients and their families undergoing radiation treatment.	2022
	CN	(A) Palliative Care	Relief of painful symptoms such as pain, dyspnea, malaise, and edema. Loss and grief care for patients and families.	1998
		(A) Cancer Pain Management Nursing	Comprehensive pain assessment, individualized care, and appropriate use of medications and pain relief.	1998
		(B) Palliative Care	Symptom management to alleviate holistic problems such as physical, psychosocial, and spiritual pain. Responding to family loss, providing grief care to their families, and improving quality of life.	2019
	CN	(A) Cancer Chemotherapy Nursing	Safe handling and proper administration of cancer chemotherapy drugs. Relief of side effects and support for self-care management.	1998
		(B) Cancer Chemotherapy and Immunotherapy Nursing	Proper administration of cancer drug therapy, risk management, and exposure control. Individualized patient education for home treatment management and response to adverse events.	2019
	CN	(A) Breast Cancer Nursing	Supporting self-care and self-determination of patients undergoing multidisciplinary treatment. Support for psychological and social problems caused by changes in body image. Prevention and management of breast cancer treatment-related complications such as lymphedema and guide breast self-examination.	2003
		(B) Breast Cancer Nursing	In addition to A courses' activities, B courses require knowledge and skills for the removal of surgical drains.	2019
	CN	(A) Radiation Oncology Nursing	Prevention, palliation, and self-care support for side effects associated with cancer radiotherapy. Provide a safe and comfortable treatment environment	2008
		(B) Radiation Oncology Nursing	Physical, psychological, and social assessment of patients undergoing radiation therapy. Symptom management and self-care support for acute and late adverse events. Management of radiation protection measures and safety control techniques to minimize medical exposure.	2019
Infection control	CNS	Infection Control Nursing	Engage in preventing individual and group infections in facilities and communities and take appropriate measures in the event of an outbreak, while providing a high standard of nursing care for patients with infectious diseases.	2006
	CN	(A) Infection Control	Implement healthcare-associated Infection surveillance practice. Assess the situation of facilities and establish an infection prevention and management system. Establishment of a system for the prevention and control of healthcare-associated infections.	1998
		(B) Infection Control	In addition to A courses' activities, B courses require knowledge and skills to administer temporary medication to those with signs of infection.	2019

WOC	CN	(A) Wound, Ostomy and Continence Nursing	Wound and excretion management such as bedsores, stoma, and incontinence. Support patient and family self-management and self-care. Total management of pressure ulcers and specialized skin care for individuals with fragile skin and individuals at risk.	1995
		(B) Wound, Ostomy and Continence Nursing	In addition to A courses' activities, B courses require the knowledge and skills to remove necrotic tissue without blood flow in the treatment of bedsores or chronic wounds and perform negative pressure closure therapy.	2019
Gerontological nursing	CNS	Gerontological Nursing	Provide a high standard of nursing care to improve the quality of life for elderly people with complex health problems, including dementia and dysphagia.	2001
	CN	(A) Dementia Nursing	Adjust the care environment and system according to each stage of dementia. Relief and prevention of behavioral psychological symptoms and social support for family members.	2004
		(B) Dementia Nursing	In addition to A courses' activities, B courses require knowledge and skills to administer anticonvulsants, antipsychotics, and anxiolytics on an ad hoc basis.	2019
Chronic care	CNS	Psychiatric Mental Health Nursing	Provide a high standard of nursing care for mentally ill patients. Provides the role of "liaison psychiatric nursing" in general hospitals.	1995
		Chronic Care Nursing	Provide a high standard of nursing care related to the prevention of lifestyle-related diseases, management of chronic diseases, health promotion, and support for people living with chronic physical and mental illness.	2003
	CN	(A) Diabetes Nursing	Disease management such as blood glucose pattern management and foot care, and support patients' medical care and lifestyle. Prevention of dialysis and support daily life according to the stage of disease.	2000
		(B) Diabetes Nursing	In addition to A courses' activities, B courses require the knowledge and skills to adjust insulin dosage.	2019
	CN	(A) Dialysis Nursing	Management of safe and comfortable dialysis treatment. Prevention of disease progression, early detection of complications and symptom management, and support for self-care. Support for self-decision-making regarding the selection, modification, and discontinuation of renal replacement therapy.	2003
		(B) Dialysis Nursing	In addition to A courses' activities, B courses require knowledge and skills to safely operate and manage hemodialysis machines in acute hemodialysis therapy.	2019
	CN	(A) Dysphagia Nursing	Assessment of feeding and swallowing function and prevention of aspiration pneumonia, choking, malnutrition, and dehydration.	2004
		(B) Dysphagia Nursing	In addition to A courses' activities, B courses require knowledge and skills to select appropriate assistance and training methods based on the results of an evaluation of feeding and swallowing function.	2019
	CN	(A) Stroke Rehabilitation Nursing	Monitoring and caring for patients to prevent critical illness. Implement early rehabilitation to maintain and promote activities.	2008
		(B) Stroke Nursing	In addition to A courses' activities, B courses require knowledge and skills to administer anticonvulsants, antipsychotics, and anxiolytics on an ad hoc basis.	2019
	CN	(A) Chronic Respiratory Nursing	Evaluation of respiratory function and management according to stable, exacerbated, and terminal stages of disease. Monitoring of respiratory symptoms and prevention of serious illness.	2010
		(B) Respiratory Nursing	In addition to A courses' activities, B courses require knowledge and skills to change settings for invasive and	2019

			noninvasive positive pressure ventilators, adjust doses of sedatives for those on ventilatory management, and wean patients from ventilators.	
	CN	(A) Chronic Heart Failure Nursing	Lifestyle adjustment and self-care support according to each life stage. Assessment and monitoring of heart failure exacerbating factors and prevention of serious illness.	2010
		(B) Heart Failure Nursing	In addition to A courses' activities, B courses require knowledge and skills to adjust the dosage of drugs (catecholamine, sodium, potassium or choler, antihypertensive drugs, glucose or electrolyte infusions, diuretics) in continuous infusion.	2019
Reproductive health, Child and family health	CNS	Child Health Nursing	Provide a high standard of nursing care in cooperation with other medical staff, to support children living with illness so that they can grow and develop healthily.	2001
		Women's Health Nursing	Provide a high standard of nursing care, including support for mothers, children, and families during the perinatal period and for women's overall health throughout the life cycle.	2002
		Family Health Nursing	Provide a high standard of physical, mental, and social nursing care to enhance their self-care ability and solve their problems independently. Support families to facilitate the patient's recovery process.	2008
		Genetics Nursing	Support decision-making for diagnosis, prevention, and treatment for medical care aimed at improving quality of life. Contribute to the establishment of the system for necessary medical treatment and care beyond generations.	2016
	CN	(A) Infertility Nursing	Provide necessary information and support self-determination for diverse choices regarding sexual and reproductive health issues to couples undergoing reproductive health care	2000
		(B) Reproductive Health Care	Guide fertility preservation and fertility control based on knowledge of sexual and reproductive functions, disorders, and risk factors.	2019
Administration	CN	Administration	Enhancing facility services by acquiring knowledge of health care and welfare policies, as well as organizational management, and establishing an educational framework while improving the workplace environment.	1998

* CN was divided into A course and B course in 2019
